# Relations between Temperament, Sensory Processing, and Motor Coordination in 3-Year-Old Children

**DOI:** 10.3389/fpsyg.2016.00623

**Published:** 2016-04-29

**Authors:** Atsuko Nakagawa, Masune Sukigara, Taishi Miyachi, Akio Nakai

**Affiliations:** ^1^School of Humanities and Social Sciences, Nagoya City UniversityNagoya, Japan; ^2^Nagoya Western Care Center for Disabled ChildrenNagoya, Japan; ^3^Department of Pediatric Neurology, Department of Pediatrics, Hyogo Children's Sleep and Development Medical Research CenterKobe, Japan

**Keywords:** temperament, sensory processing, motor coordination, effortful control, 3-year-olds

## Abstract

Poor motor skills and differences in sensory processing have been noted as behavioral markers of common neurodevelopmental disorders. A total of 171 healthy children (81 girls, 90 boys) were investigated at age 3 to examine relations between temperament, sensory processing, and motor coordination. Using the Japanese versions of the Children's Behavior Questionnaire (CBQ), the Sensory Profile (SP-J), and the Little Developmental Coordination Disorder Questionnaire (LDCDQ), this study examines an expanded model based on Rothbart's three-factor temperamental theory (surgency, negative affect, effortful control) through covariance structure analysis. The results indicate that effortful control affects both sensory processing and motor coordination. The subscale of the LDCDQ, control during movement, is also influenced by surgency, while temperamental negative affect and surgency each have an effect on subscales of the SP-J.

## Introduction

In a recent review of infant precursors of later diagnoses of Autism Spectrum Disorder (ASD) and Attention Deficit Hyperactivity Disorder (ADHD), Johnson et al. (2014) mention differences in sensory and motor functioning. They argue that a number of symptoms of ASD and ADHD may result from common mechanisms pertaining to brain adaptation or compensation in the face of early disturbances to synaptic functions. Johnson (2012) also notes that good prefrontal executive function skills may be a common protective factor across different developmental disorders, while poor executive function skills in infants at high risk may be linked to such diagnoses later in life. Consistent with this proposal, they argue that poor effortful control was found in toddlers who later received ASD and ADHD diagnoses.

The construct of effortful control emerged initially from psychometric studies using questionnaires to investigate temperament. Researchers have consistently identified neurodevelopmental disorders as being linked to specific temperament configurations (e.g., Anckarsäter et al., 2006). Temperament has been defined as a constitutionally-based set of individual differences in reactivity and self-regulation in the domains of emotion, activity, and attention, which are influenced over time by heredity, maturation, and experience (Rothbart and Bates, 2006). Factor-analytic work using parent- or self-reported behavioral questionnaires has yielded three broad factors of temperament. As distinct from the two broad factors of surgency and negative affect, a third factor, called effortful control, emerged in a series of behavioral questionnaires across the lifespan. The first two factors are concerned with emotional reactivity, while the third is related to individual differences in self-regulation and control of reactivity. Effortful control refers to the ability to inhibit a dominant response in order to perform a subdominant response, detect errors, and engage in planning. It can also be regarded as the ability to control one's actions, emotions, and attention (Rothbart, 2011).

Effortful control has been reported to undergo rapid development in childhood between the ages of 2 and 7. Developing effortful control is thought to be caused by development in the executive attention system of the brain (Posner and Rothbart, 1998). The brain's three attentional networks—alerting, orienting, and executive—are seen by these researchers as underlying achievement. Recently, it has been suggested that in the first year of life, control may primarily involve the orienting attention network, including the parietal lobe and frontal eye fields (Posner et al., 2012), the efficiency of which develops substantially during infancy (for a review, see Colombo, 2001). By 3–4 years of age, a frontal executive attention network involving the anterior cingulate and basal ganglia may take on a role of self-regulation. Temperamental effortful control could therefore be defined as the efficiency of the neural network involved in executive attention (Posner and Rothbart, 2000).

Although Johnson et al. (2014), regard temperamental effortful control as a protective or compensatory factor for neurodevelopmental disorders, relatively little attention has been paid to relations between temperament and neural markers, including poor motor skills and sensory differences. Gouze et al. (2012) point out that in studying temperament as a risk factor, researchers in the field of developmental psychopathology have focused on the emotion and attention systems and have been less concerned with the role of sensory regulation, a regulatory process operating across multiple sensory domains. Applying confirmatory factor analysis to the Child Behavior Questionnaire (CBQ) and the Short Sensory Profile (SSP), an instrument designed to screen for sensory processing difficulties in children, they attempted to replicate Rothbart's model of temperament, in which negative affect is associated with sensory reactivity and effortful control with sensory regulation. In particular, they examined whether sensory regulation was subsumed into the negative affect and effortful control factors. Their results indicate that sensory regulation is a conceptually distinct factor from the temperament factors of effortful control and negative affect.

However, Dunn (2001) suggested that sensory processing mechanisms underlie the manifestations of one's temperament and personality. Dunn attempted to integrate the four categories in her sensory processing model (low registration, sensitivity to stimuli, sensory seeking, and sensory avoiding) into the constructs of temperament or personality. She hypothesized that the sensory processing pattern of low registration (high neurological threshold and passive responding patterns) may be related to temperamental effortful control, perhaps because individuals with low registration tend to miss or take longer to respond to events in their environment and find it easier to focus on tasks of interest in distracting environments. She also noted that sensory seeking is associated with surgency while sensory avoiding and sensitivity are associated with fear and negative affect.

On the other hand, DeSantis et al. (2011) noted that relations between infant temperament and neurobehavioral measures have barely been explored and suggested a link between motor competence and temperamental attentional processes in 1-month-old infants through principle component analysis. In response, Nakagawa et al. (2015) analyzed data from 1892 infants in order to examine the above-mentioned relationship as part of the Japan Environment and Children's Study conducted by the Japanese Ministry of the Environment. Their findings are consistent with the idea that surgent tendencies should be viewed as an accelerator toward action in infants, with inhibitory tendencies such as fear (one of the subscales for negative affect) and effortful control as brakes (Rothbart, 2011). Nakagawa and Sukigara (2013) also investigated longitudinally the development of the coordination of eye and head movement by testing 12–36 month-old infants, with improvements in such coordination seemingly being accompanied by increases in the efficiency of executive attention or effortful control.

The purpose of the present study is to explore relations between temperament and two markers of neurodevelopmental disorder, namely poor motor skills, and sensory processing differences in 3-year-old children. We administrated Japanese versions of the CBQ (Rothbart et al., 2001), an instrument developed for measuring temperament in children aged 3–7, the Sensory Profile (Dunn, 1999), an instrument designed to assess a child's sensory processing patterns, and the Little Developmental Coordination Disorder Questionnaire (LDCDQ; Rihtman et al., 2011), an instrument designed to identify developmental coordination disorder at ages as young as three and four. This research was approved by the Ethics Committee of Nagoya City University. Our hypotheses are as follows. First, temperamental effortful control may regulate both motor coordination and sensory processing. Second, temperamental surgency may have an effect on motor skills. Third, some subscores in the Sensory Profile may be associated with temperamental reactivity, namely surgency or negative affect.

## Methods

### Participants

A sample of caregivers was recruited from the community in Nagoya, Japan's third largest industrial metropolis, for a previous longitudinal study that examined temperament and attention development in infancy and early childhood. Respondents were originally surveyed while visiting public health centers for their infant's routine 3-month medical examination, which is offered free of charge by municipalities in Japan. The caregivers were primarily female (98%), except for two males and one unknown. All of the caregivers were Japanese. In our previous study, we provided the questionnaires to 247 caregivers, who agreed to participate at 4 months. With some of these participants dropping off, questionnaires were mailed to the remaining participants' homes up to 24 months.

In the present study, we mailed the questionnaires to 218 caregivers who had previously stated in writing their informed consent to take part in the study. A total of 201 caregivers responded and were offered remuneration in the form of a book token worth ¥1000 (~$8). Based on the face sheet eliciting personal information covering the past 3 years about 187 infants without birth problems or disabilities, only infants carried to full term (37–42 weeks' gestation) and of normal birth weight (over 2500 g) were eligible for the current analysis, resulting in a total of 171 participants in the final sample. Average age was 38.91 months (*SD* = 1.65, Range 36–42 weeks), with 90 boys and 81 girls. The reason for leaving out premature and low birth weight infants is that these children differ from full-term and normal birth weight infants in terms of temperament on the one hand and sensory processing and motor skills on the other (Case-Smith et al., 1998; van Baar et al., 2009; Moreira et al., 2014). We needed to ensure that excluding these children would not create a spurious relationship between our substantive variables, that is, that the relationship between temperament on the one hand and sensory processing and motor skills on the other was real and not simply due to the fact that both are associated with birth status.

### Questionnaires

#### The Japanese version of child behavior questionnaire (CBQ; Kusanagi, 1993)

This 195-item parent-reported instrument is designed for children aged 3–7. Each item describes children's reactions to a number of situations. Caregivers are asked to decide whether each item is a “true” or “untrue” description of their child's reactions over the past 6 months on a scale from one (extremely untrue of the child) to seven (extremely true of the child). A total of 13 subscales yielded three broad factors: (a) surgency, which includes activity level, high-intensity pleasure, impulsivity, and shyness (which loads negatively); (b) negative affect, which includes anger, discomfort, fear, sadness, and soothability (which loads negatively); and (c) effortful control, which includes attention focusing, inhibitory control, low-intensity pleasure, and perceptual sensitivity.

#### The Japanese version of sensory profile (SP-J; Ito et al., 2013)

This original instrument (Dunn, 1999) is a 125-item questionnaire that elicits responses to sensory events in daily life. Caregivers report how frequently their child manifests a given response to particular sensory events on a 5-point Likert scale: 1 = always, 2 = frequently, 3 = occasionally, 4 = seldom, and 5 = never. Items are designed to reflect potential difficulties with sensory experiences, with a lower score reflecting poorer performance. Although, the SP-J instrument reverses the score (namely, 5 = always, 4 = frequently, 3 = occasionally, 2 = seldom, and 1 = never), the present study follows the original formulation. That is, lower scores reflect less efficient sensory processing in children's daily life. The reason why we chose to follow the original formulation is to ensure consistency in direction with the LDCDQ, in which higher scores reflect better performance (see below).

Research findings yield a conceptual model based on individual differences in neurological thresholds for stimulation [high (habituation) – low (sensitization)] and behavior response patterns (active–passive). The four quadrant scores were constructed by adding the raw scores that correspond to each item listed by Dunn (2006). These scores were low registration (15 items), sensation seeking (26 items), sensory sensitivity (20 items), and sensation avoiding (29 items). A high threshold with passive responding tendency was termed low registration, a high threshold with active responding tendency was termed sensation seeking, a low threshold with passive responding tendency was termed sensory sensitivity, and a low threshold with active responding tendency was termed sensory avoiding. Following Dunn's (2006) four quadrant scores, we constructed four scores (low registration, sensation seeking, sensory sensitivity, and sensation avoiding). That is, we summed all numerical item responses for a given quadrant grid and divided the total by the number of items receiving a numerical response.

#### The Japanese version of little developmental coordination disorder questionnaire (LDCDQ; Nakai et al., 2012)

The caregivers were given 15 items describing specific motor abilities grouped into three distinct factors: ball skills and control during movement, handwriting and fine motor skills, and general coordination, including speed of movement, fatigue, and the ability to learn new motor skills. Each factor contains five items, with parents rating their child's performance on a 5-point Likert scale (from 1 = not at all like your child to 5 = extremely like your child). When answering the questions, the parents were asked to compare the degree of coordination in their child with that of other children of the same age and gender.

## Results

### Examining the original model

A covariance structure analysis was conducted using Amos software (ver. 22) to clarify relations between sensory processing, motor coordination, and temperament (surgency, negative affect, and effortful control) in 3-year-olds. Recall that Rothbart's original model had four subscales loading on effortful control (attention focusing, inhibitory control, low-intensity pleasure, and perceptual sensitivity), four subscales loading on surgency (activity level, high-intensity pleasure, impulsivity, and shyness), and five subscales loading on negative affect (anger, discomfort, fear, sadness, and soothability). With regard to motor coordination, three subcategories of LDCDQ (control during movement, fine motor skills, and general coordination) loaded. These also yielded four scores, loading on sensory processing (low registration, sensitivity to stimuli, sensation seeking, and sensation avoiding) following Dunn's (1999) theoretical model.

**Figure 1:**
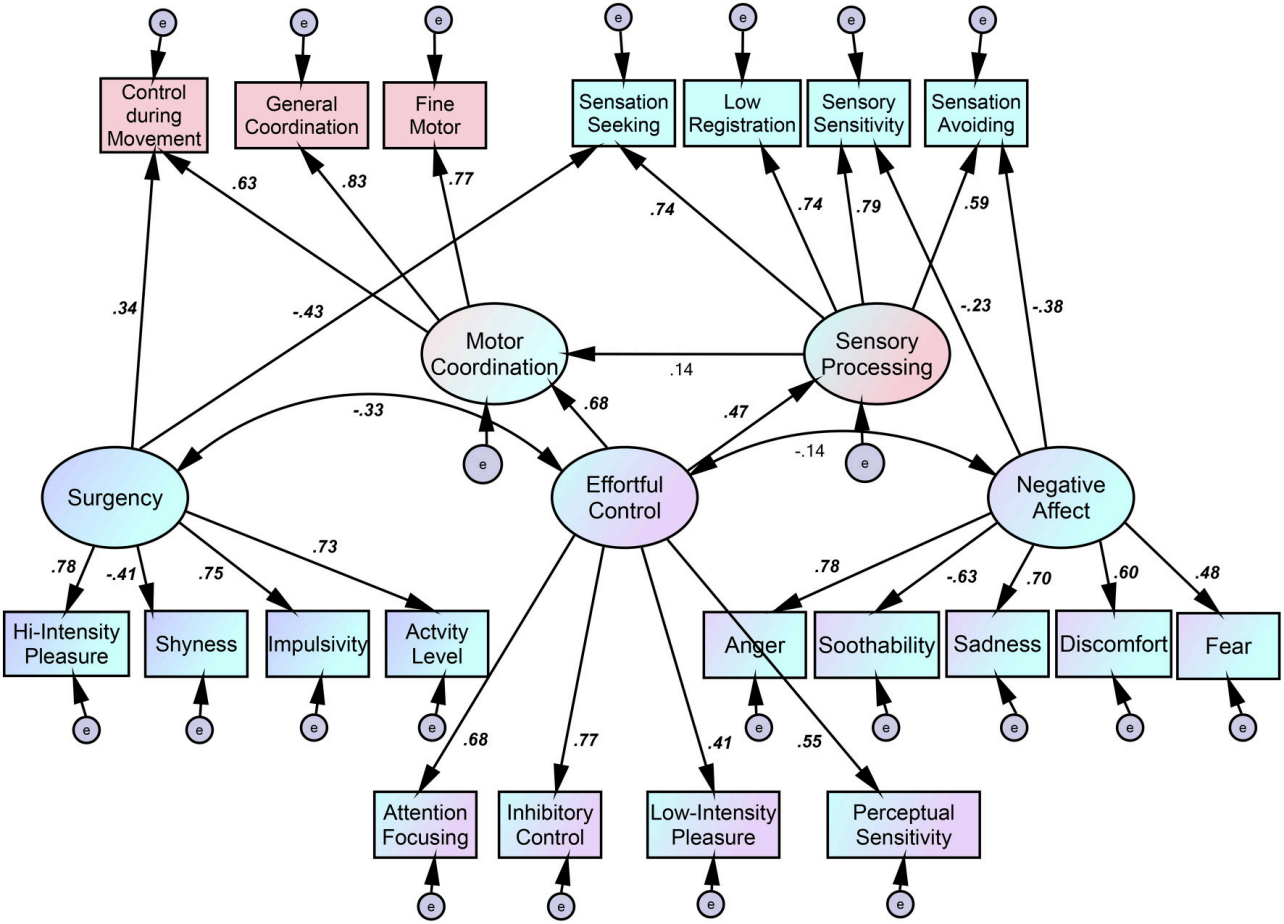
**Path diagram of causal relations between temperament, sensory processing, and motor coordination in 3-year-olds (based on the original three-factor temperament model)**. Coefficients are standardized beta values. Italic, Bold, *p* < 0.001.

The hypothesized model (Figure 1) was designed to investigate paths from the latent factor of temperamental effortful control toward the latent factors of motor coordination and sensory processing. Cross-loading from two temperamental latent factors to temperamental subscales was not allowed. Although the three factors of surgency, negative affect, and effortful control are theoretically distinct (Rothbart et al., 2001), there may be a degree of correlation between them. Regarding sensory processing, paths from the three latent temperamental factors were hypothesized, following Dunn (1999). Surgency was also thought to affect control during movement, which is the first factor in the LDCDQ and which contains a number of items related to motor control while the child is moving or an object is in motion (caught or thrown). As Nakagawa et al. (2015) found, surgency may generally facilitate adequate posture and voluntary movement in the 1 year of life. Moreover, the latent factor of sensory processing may affect the latent factor of motor coordination. However, the present model indicates a poor fit [$\chi_{\left(161\right)}^{2}$ = 465.0, *p* < 0.01, CFI = 0.791, RMSEA = 0.105].

### Examining an alternative model

**Figure 2:**
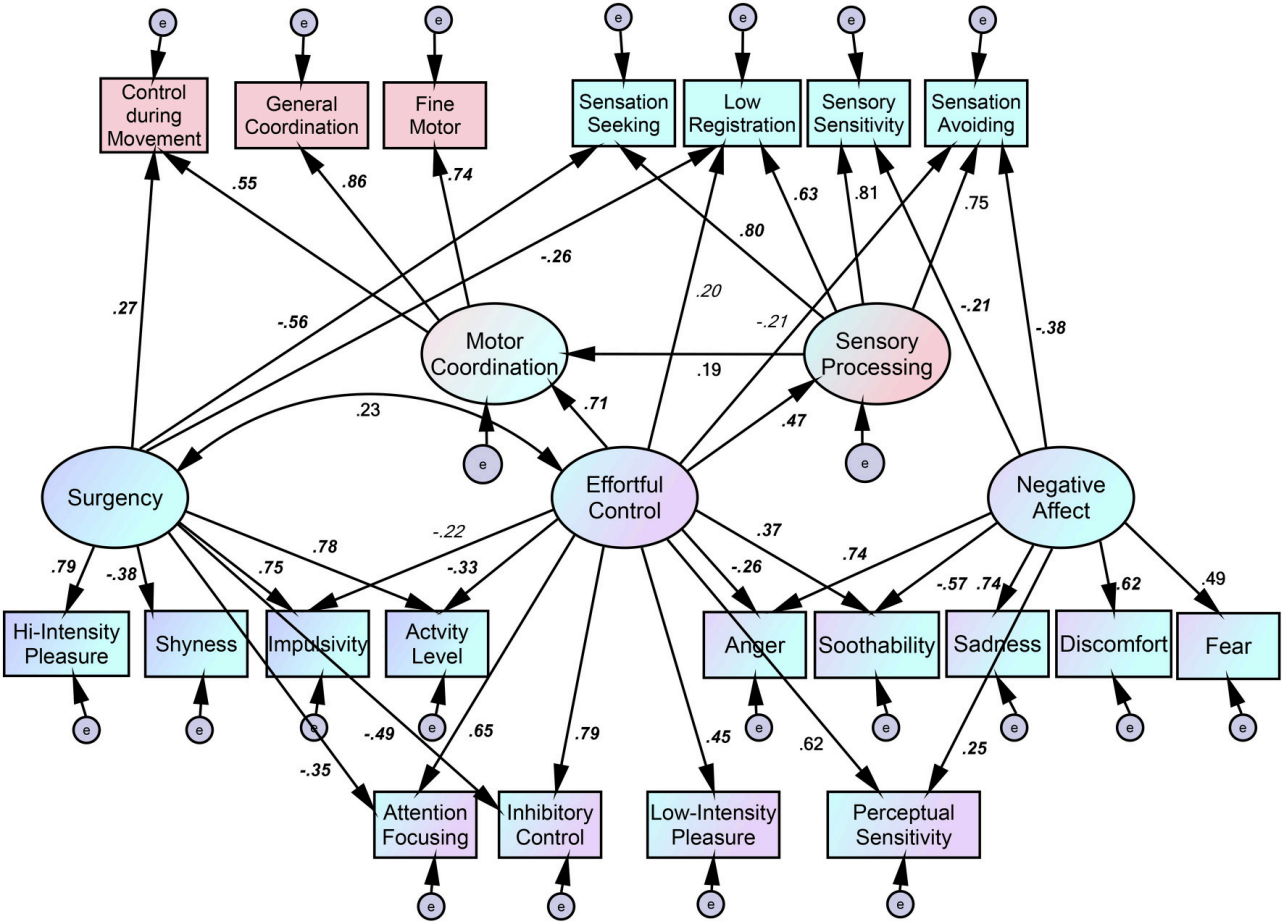
**Path diagram of causal relations between temperament, sensory processing, and motor coordination in 3-year-olds following modification indices**. Italic, Bold, *p* < 0.001; Italic, *p* < 0.01; Bold, *p* < 0.05.

The alternative model attempts to retain the original five latent factors by allowing for cross-loading of the variables being measured and follows procedures used in modification indices (Figure 2). Following Gouze et al. (2012), it consists of three scales cross-loading on both effortful control and negative affect (soothability, anger or frustration, and perceptual sensitivity). Four factors loaded on both effortful control and surgency (impulsivity, activity, inhibitory control, and attentional focusing). These are not inconsistent with previous results (Rothbart et al., 2001). Concerning sensory processing, the path from effortful control to sensation avoiding was added because neuroticism, which in adult personalities corresponds to sensory avoiding (Dunn, 2001), and effortful control are negatively related to it (Rothbart et al., 2000). Another path from surgency to low registration was provided. As low registration is equivalent to conscientiousness (Dunn, 2001), surgency includes the characteristic of impulsivity and should correlate negatively with conscientiousness (Grist and McCord, 2010), a characteristic that can “range from organized, thorough, and responsible to careless, disorderly, and slipshod” (Rothbart, 2011, p. 193). This alternative model shows an adequate fit [$\chi_{\left(152\right)}^{2}$ = 314.3, *p* < 0.01, CFI = 0.888, RMSEA = 0.079].

### Relations between sensory profile, motor coordination, and temperament

We found that effortful control, or the efficiency of the executive attention network, may influence both the latent factors of motor coordination and sensory processing, with *b* weights of 0.71 and 0.47, respectively. Effortful control also shows an effect on the subscales of sensory processing, namely low registration and sensation avoiding, with *b* weights of 0.20 and –0.21, respectively. Moreover, the latent factor of surgency influences the LDCDQ subscale of control during movement, with a *b* weight of 0.27, and also contributes to the subscales of sensory processing, namely sensation seeking and low registration, with *b* weights of –0.56 and –0.26, respectively. Similarly, the latent factor of negative affect has a negative effect on the subscales of sensory processing, namely sensation avoiding and sensory sensitivity, with *b* weights of –0.38 and –0.21, respectively.

To test the significance of three indirect effects, the RMediation software (Tofighi and MacKinnon, 2011) was used to examine 95% confidence intervals (CIs) for each of the two-path indirect effects. For the indirect effect of effortful control on low registration via sensory processing, the 95% CI was [0.05, 0.138]. For the indirect effect of effortful control on sensation avoiding via sensory processing, the 95% CI was [0.077, 0.209], while for the indirect effect of effortful control on motor coordination via sensory processing, the 95% CI was [0.016, 0.325]. The results suggest that effortful control has significant and indirect effects on low registration and sensation avoiding via sensory processing as well as a significant and indirect effects on motor coordination via sensory processing.

## Discussion

This study addressed relations between sensory processing, motor coordination, and temperament early in life. Following a covariance structure analysis, both motor coordination and sensory processing, which have been reported to be associated with behavioral and neural markers of neurodevelopmental disorders, appear to be influenced by effortful control. This is consistent with the view that high temperamental effortful control, or high efficiency of the executive function, may compensate for atypicalities in other brain systems early in life (Johnson, 2012).

Our model shows only an adequate fit. Moreover, unlike Rothbart's original model, which suggests that three temperamental factors, namely effortful control, surgency, and negative affect are distinct, our model includes three scales cross-loading on effortful control and negative affect and four scales cross-loading on effortful control and surgency. However, we were not surprised to find that the fit was not especially good for the CBQ as this instrument was not designed with a specific structure in mind, with the structure emerging instead in an exploratory factor analysis. As indicated in the original paper (Rothbart et al., 2001), the model achieved only a satisfactory fit after researchers altered it with reference to modification indices. In addition, in that study, there were differences between the structures identified in the US and Chinese samples and between those identified in 3-year-olds and older children. Through a confirmatory factor analysis conducted in order to reproduce the original Rothbart model for negative affect and effortful control, Gouze et al. (2012) also reported that the model produced a poor fit without modification indices.

Moreover, Gouze et al. (2012) found that sensory regulation stands alone as a factor independently of negative affect and effortful control, even though established models of temperament include sensory items within the negative affect and effortful control factors. However, as these researchers obtained a better fit for the model with reduced item contamination (i.e., after elimination of items), there may be a conceptual problem, as the authors note. On the other hand, Dunn (2001) hypothesized a correlation between temperament and patterns of sensory processing. In her model, sensory seeking is linked to surgency in childhood, sensory avoiding is associated with negative affect, and sensory sensitivity is associated with irritability and anger, while low registration (high thresholds and passive responding tendency) is hypothesized to be undistracted by other stimuli and to enable task performance. Dunn speculates that there may be a correlation between low registration and effortful control. The present results are consistent with the integrative model of infant behavior in DeSantis et al. (2011), which proposes two significant relationships, one between sensory processing with low threshold to sensory stimuli and Rothbart's negative emotionality, and another between sensation seeking and low registration (both in high threshold quadrant) and Rothbart's surgency. These researchers thus suggest combining sensory processing data with the temperament and personality constructs across disciplines and then applying this knowledge in practice.

Since, we were interested in relations between effortful control and sensory processing or motor coordination, we were not particularly concerned with constructing a model with good fit and reduced item contamination. Nor did we wish to alter the original factor structures of temperament. According to the adequate fit model shown in Figure 2, we speculate that effortful control plays a role in regulating sensory processing and motor coordination through each latent factor. As expected, effortful control also directly influences sensory avoiding in a negative direction. A low score on sensation avoiding means that the child engages more frequently in avoiding behaviors. As some avoiding behaviors in these items reflect modulation of movements, a lower score on sensation avoiding may be linked to a higher score on effortful control.

Concerning motor coordination, our results met our expectations. The latent factor of this dimension was strongly influenced by effortful control, and the subscale of control during movement was strongly associated with surgency. Children high in smiling and laughter as well as surgency may rapidly engage in an activity. Children's enthusiasm, shown in interest, pleasure, and motivation to learn as well as in engagement seen as attention and persistence may be driven by their positive and approaching tendencies (Rothbart, 2011). A number of items related to motor control while the child is moving or an object is in motion, labeled control during movement, may be linked to deriving pleasure from the action itself.

In addition, Figure 2 indicates that sensory processing makes a contribution to motor coordination. This may be consistent with the view that coordinated movement depends on integrating sensory information or that a history of sensory disturbance may be sought as a possible factor in impairing coordination (Gibbs et al., 2007). In this view, adaptive behavior responsiveness, learning, and coordinated movement are considered products of efficient reception and integration of incoming sensory signals (Bundy et al., 2002).

A limitation of our study is that our sample size was somewhat small, and greater statistical power would be provided by a larger sample size. In addition, to be included in this study, infants had to have been carried to full term (37–42 weeks' gestation) and be of normal birth weight (over 2500 g). If assessment tools used for measuring sensory processing and motor coordination are not designed to screen for problems but rather for typical sensory functions, we may arrive at different results. Future investigations should be based not only on parent-reported questionnaire data but also on laboratory observations and naturalistic home observations as each of these approaches has advantages as well as disadvantages (Rothbart, 2011). Moreover, as effortful control is thought to develop rapidly in children between the ages two and seven, its development may well-mediate or moderate the relationship between early signs of these atypicalities and later signs of them, though this would require a longitudinal study. Thus, our model remains work in progress.

Despite these limitations, our study proposes a unique integrative model of 36-month toddlers that could incorporate temperament, sensory processing, and motor coordination. Recent neuroimaging findings showing that the same region of the anterior midcingulate cortex is engaged in motor control and pain processing (Misra and Coombes, 2015) may constitute supporting evidence. Although these constructs have been taken into consideration in different ways within the disciplines of developmental psychology, occupational therapy, and behavioral pediatrics (DeSantis et al., 2011), recent developments in cognitive neuroscience make it possible for us to take up these relations for examination. The results of our study should also be examined longitudinally in order to demonstrate developmental changes before early adolescence. Further research is required if we are to determine the validity of our findings for understanding child development and for suggesting significant implications for assessment and intervention aimed at improving the moderating factor of effortful control.

## Author contributions

AtN conceived and designed the study. AkN organized the administration of questionnaires. TM contributed to data collection. MS performed the statistical analyses and helped draft the manuscript. TM and AkN contributed to the interpretation of the data and helped to revise the manuscript. All authors read and approved the final draft of the manuscript.

### Conflict of interest statement

The authors declare that the research was conducted in the absence of any commercial or financial relationships that could be construed as a potential conflict of interest.
